# Imaging dynamic fingerprints of competing E2 and S_N_2 reactions

**DOI:** 10.1038/s41467-017-00065-x

**Published:** 2017-06-21

**Authors:** Eduardo Carrascosa, Jennifer Meyer, Jiaxu Zhang, Martin Stei, Tim Michaelsen, William L. Hase, Li Yang, Roland Wester

**Affiliations:** 1 Institut für Ionenphysik und Angewandte Physik, Universität Innsbruck, Technikerstrasse 25/3, Innsbruck, 6020 Austria; 2 School of Chemistry and Chemical Engineering, Harbin Institute of Technology, Harbin, 150001 P. R. China; 3 Department of Chemistry and Biochemistry, Texas Tech University, Lubbock, TX 79409 USA

**Keywords:** Reaction mechanisms, Chemical physics, Reaction kinetics and dynamics

## Abstract

The competition between bimolecular nucleophilic substitution and base-induced elimination is of fundamental importance for the synthesis of pure samples in organic chemistry. Many factors that influence this competition have been identified over the years, but the underlying atomistic dynamics have remained difficult to observe. We present product velocity distributions for a series of reactive collisions of the type X^−^ + RY with X and Y denoting the halogen atoms fluorine, chlorine and iodine. By increasing the size of the residue R from methyl to tert-butyl in several steps, we find that the dynamics drastically change from backward to dominant forward scattering of the leaving ion relative to the reactant RY velocity. This characteristic fingerprint is also confirmed by direct dynamics simulations for ethyl as residue and attributed to the dynamics of elimination reactions. This work opens the door to a detailed atomistic understanding of transformation reactions in even larger systems.

## Abstract (web summary)

The competition between chemical reactions critically affects our natural environment and the synthesis of new materials. Here, the authors present an approach to directly image distinct fingerprints of essential organic reactions and monitor their competition as a function of steric substitution.

## Introduction

Base-induced elimination (E2) and bimolecular nucleophilic substitution (S_N_2) are probably the two most fundamental exchange reactions in organic chemistry^1^. Both processes are stereo-specific, occur in a concerted way and always appear in competition with each other. Due to the ubiquity and importance of this competition, immense experimental and theoretical effort has historically been devoted to investigate both the intrinsic reactivity and atomistic dynamics of these reactions^2–6^. In the past years, new experimental and theoretical developments have provided detailed insight into bimolecular nucleophilic substitution reactions between monatomic anions and methyl halides (X^−^ + CH_3_Y)^7–14^. While early studies already revealed non-statistical reaction kinetics and dynamics and thereby showed that such systems cannot be described using common statistical models such as Rice–Ramsperger–Kassel–Marcus (RRKM) or phase space theory^15, 16^, very recent investigations have theoretically postulated quantum state specificity in S_N_2 reactions^17, 18^. Furthermore, new mechanisms beyond the classical collinear attack have been revealed and characterized^8, 19^. All these advances reflect that the understanding of the underlying mechanistic features of nucleophilic substitution is a complex and ongoing subject of research.

**Fig. 1:**
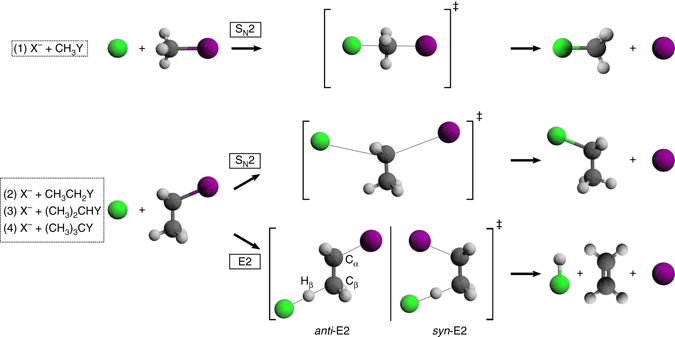
Schematic representation bimolecular nucleophilic substitution (S_N_2) and elimination (E2) mechanisms. For S_N_2 the back-side attack TS is shown, whereas for E2 both the *anti-* and the *syn*-TSs are presented. Systems (1)–(4) represent the studied reactions with stepwise substituted alkyl halides

One of the most relevant questions related to the dynamics of S_N_2 processes is how substitution of the halogenated central carbon atom (*α*-carbon) affects both the dynamics and reactivity of such systems. The addition of bulky substituents is supposed to hinder the well-known Walden inversion and promote the E2 reaction. Such an elimination process is assumed to occur via an anionic attack on a *β*-carbon-bonded hydrogen atom in periplanar orientation with respect to the *α*-carbon-Y bond (see Fig. 1). This H-atom can be placed in an *anti*- or *syn*-configuration with respect to the leaving halogen atom, the latter geometry being energetically less favourable^20, 21^.

Studies related to S_N_2 and E2 processes have analyzed how the characteristics of the attacking and leaving anions, the substituents, and the environment influence the reaction probability between S_N_2 and E2 reactions^22–24^. By studying complex reactive systems where both substitution and elimination can occur, many challenges arise for experiment and theory: In computations the high complexity introduced by adding substituents to the halogenated hydrocarbon makes atomistic simulations very demanding. To date, most theoretical predictions about the ratio between elimination and substitution have been indirectly inferred from stationary point electronic structure calculations^25, 26^. In particular, many studies have dealt with the influence of the degree of methyl-substitution on the height of the potential energy barriers for E2 and S_N_2 reactions as an indirect estimation of the preference for a specific reaction^27, 28^. However, these calculations cannot account for dynamical effects such as the influence of the impact parameter, the role of reactant reorientation during the collision, the specific atomistic reaction mechanisms, or the energy partitioning^16^. These aspects very likely exercise a significant influence on the preference for specific pre-reaction complex geometries and affect the dynamics and relative efficiency of both reactions.

Experimentally, single-collision studies have become very useful in order to understand the intrinsic mechanistic behavior of organic reactions in a solvent-free environment^29, 30^. However, disentangling S_N_2 and E2 reaction products by conventional mass spectrometry is challenging considering that both reactions yield the same ionic species, as illustrated in Fig. 1. Over the past decades, many investigations have provided indirect mass spectrometric estimations of the branching ratio between S_N_2 and E2 for a variety of systems: deuterium kinetic isotope effects have been employed as a qualitative measure of the tendency of a specific reaction towards substitution or elimination^24, 31^. Although these studies provide very useful kinetic information, they lack a direct quantitative determination of the competition between substitution and elimination. Other approaches have shown to be better suited to obtaining branching ratios between S_N_2 and E2. Brauman and coworkers have measured the reaction rate in systems where the S_N_2 ion-molecule complex undergoes an internal proton transfer leading to a different ion as the S_N_2 product^32^. Gronert and coworkers have made use of nucleophilic dianions that form distinct elimination and substitution products upon reaction^2, 23^. Although the latter method allows one to obtain direct ratios, the presence of bulky dianions introduces steric effects that very likely influence the dynamics and kinematics of such reactions. In this work, we are able to directly image the transition from S_N_2 to E2 as a function of methyl-substitution and identify, both experimentally and theoretically, an atomic-level mechanism intrinsic to E2 reactions.

## Results

### Product ion velocity distributions

Using an ion-molecule reactive scattering apparatus^29, 33^ that combines crossed beam scattering^34^ and three-dimensional velocity map imaging (VMI)^35^ techniques (experimental details are presented in the Methods section) we have obtained energy- and angle differential cross-sections for a series of reactions of types (1)–(4), as indicated in Fig. 1. Three different X–Y combinations (F–Cl, F–I and Cl–I) have been studied in order to rationalize the influence of anion nucleophilicity/basicity and leaving group ability on the dynamics and relative efficiency of the E2 and S_N_2 pathways. For each of these combinations reactive scattering experiments have been performed on reactions with four increasingly methylated neutral species (CH_3_Y, C_2_H_5_Y, ^i^C_3_H_7_Y and ^t^C_4_H_9_Y) that represent the transition from primary to tertiary alkyl halides. The corresponding reaction enthalpies are listed in Table 1.

**Table 1:** Substitution and elimination reaction enthalpies (Δ_r_H°) for all investigated systems, calculated using tabulated formation enthalpies^48, 49^

Reaction	Δ_r_H° (S_N_2) [eV]	ΔrH° (E2) [eV]
F^−^ + CH_3_Cl	−1.35	–
F^−^ + C_2_H_5_Cl	−1.39	−0.91
F^−^ + ^i^C_3_H_7_Cl	−1.32	−0.90
F^−^ + ^t^C_4_H_9_Cl	−1.28	−0.90
F^−^ + CH_3_I	−1.84	–
F^−^ + C_2_H_5_I	−2.12	−1.58
F^−^ + ^i^C_3_H_7_I	−2.00	−1.58
F^−^ + ^t^C_4_H_9_I	−1.65	−1.58
Cl^−^ + CH_3_I	−0.55	–
Cl^−^ + C_2_H_5_I	−0.67	+0.07
Cl^−^ + ^i^C_3_H_7_I	−0.68	+0.07
Cl^−^ + ^t^C_4_H_9_I	−0.37	+0.39

**Fig. 2:**
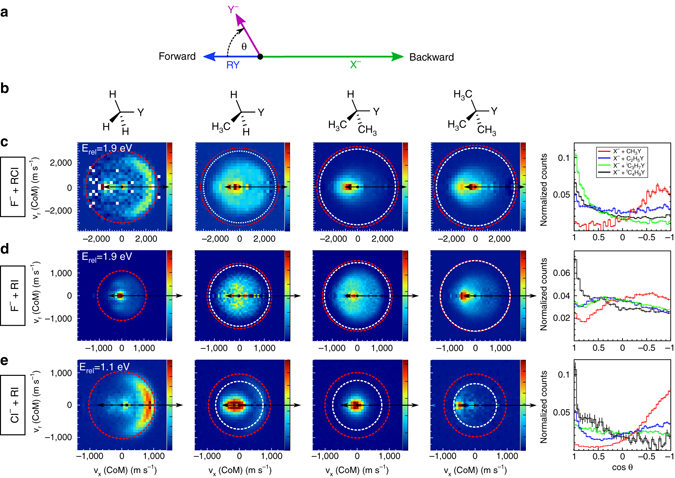
Experimental centre-of-mass product velocity and scattering angle distributions. **a** Scheme illustrating the directions and velocities of the rectants in the centre of mass frame and defining forward and backward scattering, as well as the scattering angle *θ*. **b** Chemical structures of the neutral reactants. **c–e** Velocity map images for four reactions of type X^−^ + RY (R=CH_3_, C_2_H_5_, ^i^C_3_H_7_, ^t^C_4_H_9_): **c**: F^−^ + RCl; **d**: F^−^ + RI; **e**: Cl^−^ + RI. Each histogram consists of a sum of about 50,000 product ion events. For each nucleophile-leaving group combination, the right panel illustrates the corresponding scattering angle distribution as function of methyl substitution of the neutral reactant, where the error bars depict the wighted statistical errors for each histogram bin. A clear transition from backward to forward scattering dynamics is observed in all three cases. The energetic limits for the S_N_2 and E2 channels are marked by red and white dashed circles, respectively. The experimental result for F^−^ + CH_3_Cl (upper left image) has been adapted from a recent publication^13^

Figure 2 presents the angle- and energy differential cross-sections of all tabulated reactions. Briefly, each image represents an accumulated product ion velocity distribution in the centre-of-mass frame. The dashed circles represent the energetic limits for the substitution (red) and elimination (white) reactions, respectively. Fig. 2c presents the centre-of-mass velocity distributions of ^35^Cl product ions from reactions between F^−^ and increasingly substituted alkyl chlorides at a collision energy of 1.9 eV. For F^−^ + CH_3_Cl dominant backward scattering of the Cl^−^ product ion is observed with respect to the incoming CH_3_Cl velocity, while scattering into the forward hemisphere and isotropic scattering barely contribute to the velocity distribution. This backward scattering feature corresponds to the commonly assumed collinear substitution mechanism under Walden inversion and has already been reported in previous studies on this and other similar systems^8, 13, 36^. Both backward and forward scattering are present in the reaction F^−^ + C_2_H_5_Cl, whereby backward scattering events appear at velocities considerably lower than in F^−^ + CH_3_Cl, indicative of a higher degree of energy partitioning among the larger number of C_2_H_5_Cl rovibrational modes. Ions produced from F^−^ + ^i^C_3_H_7_Cl and F^−^ + ^t^C_4_H_9_Cl are predominantly forward scattered and no backward scattering is observed, as can be extracted from the scattering angle distributions on the upper right panel of Fig. 2.

Fig. 2d shows product I^−^ ion images from reactions between F^−^ and stepwise substituted alkyl iodides. As discussed in a previous work, the reaction between F^−^ and CH_3_I features dominant indirect as well as direct stripping dynamics^37^. The reaction F^−^ + C_2_H_5_I presents a combination of forward, backward and sideways scattered events, whereas both F^−^ + ^i^C_3_H_7_I and F^−^ + ^t^C_4_H_9_I show dominant forward scattering of the I^−^ product. There are clear differences between the velocity distributions of F^−^ + CH_3_Cl and F^−^ + CH_3_I, which was recently shown to arise from a substantially different relative orientation of the respective reactants^13^. In contrast, we observe forward scattering of the leaving Cl^−^ or I^−^ ion in reactions with ^t^C_4_H_9_Y. As such a scattering pattern has never been observed for nucleophilic substitution reactions, we assign this feature to the atomic-level mechanism of base-induced elimination. This is in line with more indirect previous findings that ^t^C_4_H_9_Y reactions occur via E2 due to the strong steric hindrance at the *α*-carbon^31^.

In order to confirm the inherent character of this mechanism irrespective of the attacking anion, additional experiments between Cl^−^ and different alkyl iodides have been performed. The resulting centre-of-mass product velocity distributions are presented in Fig. 2e and again show a clear transition from dominant backward scattering in Cl^−^ + CH_3_I toward a forward scattering mechanism in reactions with ^t^C_4_H_9_I, as clearly illustrated in the lower right scattering angle distribution of Fig. 2. For Cl^−^ + C_2_H_5_I a high fraction of backward scattered events show velocities above the kinematic limit of the E2 reaction, unambiguously indicating nucleophilic substitution events. In contrast to the nucleophile-leaving group combinations discussed before, backward scattered events are still observed both in reactions with C_2_H_5_I and ^i^C_3_H_7_I. This fact can be explained by the lower proton affinity of Cl^−^ and consequently by the smaller tendency of undergoing E2 reactions at increasing CH_3_-substitution. The observed mechanistic transition as a function of methylation is very similar to the reactions with F^−^ ions and reinforces the indication that scattering into the forward hemisphere is a mechanistic fingerprint of E2 reactions.

### Reaction mechanisms and atomistic dynamics

**Fig. 3:**
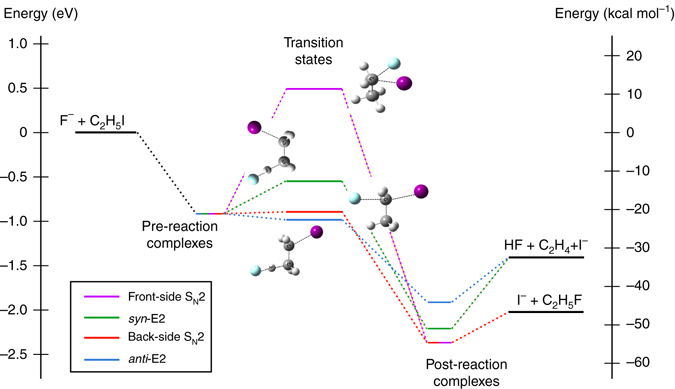
Schematic representation reaction energy profiles for E2 and S_N_2 pathways in F^−^ + CH_3_CH_2_I. The calculations have been carried out at the M06/ECP/d level of theory. The optimized TS structures are shown for all four reaction mechanisms. The indicated energies include zero-point energy (ZPE) corrections

To complement the experimental findings, the main atomic-level mechanisms involved in the F^−^ + C_2_H_5_I reaction were investigated by quasi-classical trajectory direct chemical dynamics simulations^38^ at a relative collision energy of 1.9 eV (see Simulations section for details on the theoretical method). The resulting potential energy profiles for the different S_N_2 and E2 pathways are shown in Fig. 3; they have been reported in detail in^39^. Both S_N_2 and E2 exhibit strongly bound pre- and post-reaction complexes separated by central transition states (TSs). Three of the TSs are lower in energy than the reactants, while a significant potential energy barrier of 0.49 eV is obtained for the front-side S_N_2 pathway. However, this barrier is much less than the collision energy of 1.9 eV. The back-side S_N_2 and *anti*-E2 TSs are very similar in energy and lie substantially below the *syn*-E2 TS.

**Fig. 4:**
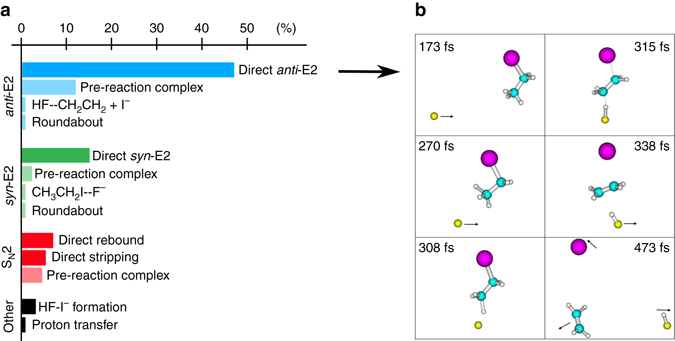
**a** Branching ratio of the different mechanisms of S_N_2 and E2 reactions in F^−^ + C_2_H_5_I at 1.9 eV. Direct reaction pathways exhibit a dark colour, whereas all indirect mechanisms are marked by lighter colours. **b** Atomistic dynamics of a typical trajectory for the direct *anti*-E2 mechanism. The numbers indicated in the upper area of the cartoons correspond to the trajectory time in femtoseconds (10^−15^ s)

Fig. 4a shows the obtained mechanisms and their respective branching ratio from the reactive trajectories. Base-induced elimination is by far the dominant channel, representing over 80% of all reactive trajectories. For the E2 pathway both direct and indirect mechanisms are identified, the former having the largest probability. A high fraction of *anti*-E2 trajectories proceed via a direct pathway. Representative snapshots of such a trajectory are presented in Fig. 4b. The mechanism proceeds via an *anti*-periplanar TS geometry followed by simultaneous formation of HF and I^−^. As shown in the cartoon, the anion usually approaches at large impact parameters and abstracts a *β*-hydrogen without modifying its original direction. Such an atomic-level mechanism is very similar to the stripping dynamics observed in several X^−^ + CH_3_Y reactions^12^. All E2 reactive trajectories were found to depend on the initial relative orientation of the anion and the *β*-hydrogen, as no rotation of the CH_3_ group was observed. This is expected given a CH_3_ rotational barrier of 0.15 eV, which cannot be surmounted under the internal temperatures of the neutral reactant (see Methods section).

**Fig. 5:**
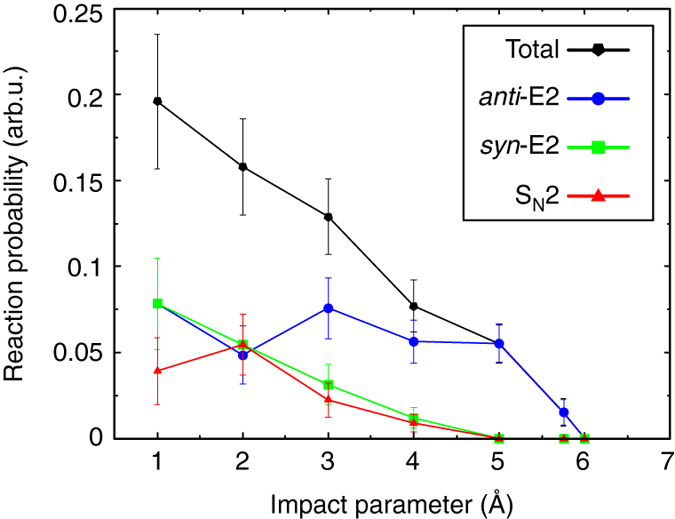
Reaction probability of the three main reaction mechanisms as a function of impact parameter. The relative collision energy is set to 1.9 eV. The vertical error bars represent the statistical uncertainty in the reaction probability at each impact parameter, computed from the square root of the number of reactive events

A smaller fraction of trajectories is found to follow indirect elimination dynamics, mainly through the formation of a long-lived ion-dipole complex. Some trajectories follow an alternative pathway and form the alternative post-reaction complexes [HF–C_2_H_4_] + I^−^ or [CH_3_CH_2_I–F^−^]. The roundabout pathway^8^ also participates in the indirect E2 mechanism. In addition, both dihalide formation and proton transfer reactions were observed after overcoming the *syn*-E2 and *anti*-E2 TSs, respectively. The formation of these products has been recently reported to be efficient in F^−^ + CH_3_I^40^ and is also observed to a minor extent in the current experiments. As also found for a variety of X^−^ + CH_3_Y reactions^12^, the S_N_2 pathway occurs via direct rebound, direct stripping and indirect mechanisms. Average direct *anti*-E2 reactions finish in about 500 fs (see Fig. 5). Nearly equivalent average trajectory times of 500–700 fs are obtained for the direct *syn*-E2 and S_N_2 pathways. The time for indirect elimination trajectories lies in the range of 1000–1200 fs.

In order to elucidate possible differences between the reaction dynamics of S_N_2, *anti*-E2 and *syn*-E2 reactions we analyzed the opacity function of F^−^ + CH_3_CH_2_I. The results are shown in Fig. 5. Overall, a decrease of the reaction probability with increasing impact parameter *b* is observed. When considering each of the three main pathways separately, the probability for anti-elimination shows no marked dependence on the impact parameter and remains high even at high values of *b*. Such high relative reaction probabilities at high impact parameters have been observed for direct stripping trajectories in X^−^ + CH_3_Y reactions, which usually lead to forward and sideways scattering of the product ion^10, 37^. In contrast to this, the reaction probabilities for the *syn*-E2 and S_N_2 pathways drastically decrease at large impact parameters. These findings are in accordance with the geometrical character of the respective TSs, where only the *anti*-E2 TS shows a relatively extended and relaxed structure, where the anion can easily abstract the *β*-hydrogen even approaching from large distances.

To further compare with experiments, the product scattering angle distribution for each of the E2 reactive pathways has been analyzed. Table 2 summarizes the relative fraction of product ions scattered into different angle regions for both the *anti*-E2 and *syn*-E2 mechanisms. Forward and backward scattering along the centre of mass are represented by *θ* = 0° and *θ* = 180°, respectively. Both *anti*-E2 and *syn*-E2 trajectories show a marked preference for sideways- and forward scattering over backward pathways. Overall, the theoretical findings agree very well with the experimental product scattering angle and reinforce the assignment of a stripping-like forward scattering mechanism inherent to E2. Regarding the energy partitioning, simulations predict that 40.9 and 33.3% of the initially available energy is transfered into product ion translational motion in direct *anti*-E2 and *syn*-E2 reactions, respectively. These fractions are very similar to the experimentally obtained value of 35.1%, which is given by the product ion mean translational energy with respect to the kinematic limit for the E2 channel. As *syn*-E2 reactions occur at lower impact parameters it is expected that more energy is transfered into internal degrees of freedom if compared with *anti*-E2 processes. Thus, these results are in line with the impact parameter analysis.

**Table 2:** Fraction of total reactive trajectories as a function of product ion scattering angle for the different *anti*-E2 and *syn*-E2 mechanisms

Reaction mechanism	Scattering angle			
	0–45°	45–90°	90–135°	135–180°
*anti*-E2 direct	0.14	0.24	0.14	0.055
*anti*-E2 indirect	0.07	0.055	0.02	0.02
*syn*-E2 direct	0.07	0.045	0.035	0.03
*syn*-E2 indirect	0.035	0.01	0.035	–
Total (theory)	**0.32**	**0.35**	**0.23**	**0.10**
Total (experiment)	**0.14**	**0.40**	**0.34**	**0.11**

## Discussion

The differential scattering cross-sections for a series of reactions between monatomic anions and increasingly methyl-substituted alkyl halide molecules have been measured. Strong similarities between the reactions of different anions with tert-butyl halides clearly indicate the presence of a mechanism inherent to E2 reactions that leads to forward scattered products. By monitoring the effect of methylation, direct imaging of the transition from substitution to elimination, characterized by the change from backward scattering to scattering into the forward hemisphere, is observed. Direct chemical dynamics trajectory simulations on F^−^ + C_2_H_5_I show *anti*-E2 as the dominant reaction channel. Both the significant reaction probability for this channel at high impact parameters and the clear preference for forward and sideways scattering of the product ion agree with the experimental findings. This combined experimental and theoretical reaction dynamics study provides evidence of the intrinsic mechanisms of bimolecular elimination and introduces a method to monitor the competition between S_N_2 and E2 reactions and its dependence on steric hindrance. The present results show that the concerted atomistic dynamics of molecular reactions can be unraveled even when a considerable number of atoms are involved. These investigations pave the way for future studies of the dynamics of bond formation and cleavage in other complex polyatomic systems such as aromatic ring formation and for the active control of the branching between different reaction mechanisms by electric fields or optical excitation.

## Methods

### Experiment

The reactive scattering experiments were carried out using a combination of crossed beams and VMI^29, 33^. The whole experiment is operated at 20 Hz repetition rate. The reactant ions are produced by dissociative electron attachment of the given precursor in a plasma discharge source. F^−^ and Cl^−^ are generated from mixtures of 10% NF_3_ in Argon and 5% CH_3_Cl in Argon, respectively. The ion species are extracted, mass selected and guided into an octupole radiofrequency ion trap via a Wiley–McLaren type time-of-flight spectrometer^41^. Here, the kinetic energy spread of the ions is minimized through non-reactive collisions with pulsed N_2_ buffer gas and spatial confinement of the ion cloud using three spherical shaping electrodes. The extracted ion beam is decelerated and crossed with a supersonic neutral molecular beam at a relative collision angle of 60° in the interaction region of the VMI spectrometer. The molecular beam consists of a low concentration of reactant gas seeded in helium. Some of the ion-molecule collisions are reactive and induce either nucleophilic substitution or elimination. The product ions are extracted normal to the scattering plane by pulsing on the electrodes of the VMI spectrometer. The ion detection scheme combines time- and position monitoring. The spatial component is detected by a combination of two multi-channel plates in chevron configuration attached to a phosphor-screen, whose light origin is recorded by a charge-coupled device camera. In addition, a photomultiplier records the arrival time of this light, therefore achieving a three-dimensional velocity detection. The raw laboratory images are then transformed into velocity maps in the centre-of-mass frame. Due to the cylindrical symmetry of the scattering process around the relative velocity axis, the full velocity and angular information can be displayed in a two-dimensional way. Each image shown in this work contains ~50,000 events.

In order to obtain energy differential cross sections, the relative collision energy is varied by setting the ions’ translational energy. Typical kinetic energy spreads achieved in the experiment are in the range of 130–200 meV (full-width at half-maximum). The concentration and backing pressure of the neutral reactant are optimized in order to avoid possible clustering. The pulsed valve is heated to avoid condensation of the nozzle. The velocity and translational temperature of the neutral molecules are obtained by electron impact ionization of the supersonic beam and imaging of the resulting non-fragmented ions. We obtain translational temperatures of 160–170 K, which are considered an upper limit due to the momentum transfer of the impinging high-energy electrons. The rotational temperature is assumed to lie in the same range, whereas a temperature between 165 and 345 K (temperature of the heated valve) is assumed for the vibrations, which are predicted to cool less efficiently during supersonic expansion.

### Simulations

The potential energy profile of the reaction between F^−^ and C_2_H_5_I has been characterized using the M06 density functional method using an effective core potential (ECP)/d basis set^42^. This method has been shown to give the best agreement with the CCSD(T)/pseudo-potential (PP)/t benchmark, as compared to other density functionals^39^. For the ECP/d, Dunning and Woons aug-cc-pVDZ basis set is used for the H, C and F atoms. For iodine, the Wadt and Hay ECP is used for the core electrons and a 3*s*,3*p* basis set for the valence electrons, which is augmented by a *d*-polarization function with a 0.262 exponent, and *s*, *p* and *d* diffuse functions with exponents of 0.034, 0.039 and 0.0873, respectively. For the PP/t basis set, the Peterson aug-cc-pVTZ basis with a PP is used for iodine and the aug-cc-pVTZ basis for all other atoms.

Chemical dynamics simulations were performed by ab initio direct dynamics at the M06/ECP/d level of theory. For this purpose, the VENUS general chemical dynamics computer program was used^43^ and interfaced^44^ to the NWChem electronic structure computer program^45^. Including zero-point energy (ZPE) corrections, this theory gives reaction exothermicities of −46.5 and −32.4 kcal mol^−1^ for the S_N_2 product C_2_H_5_F + I^−^ and the E2 product CH_2_=CH_2_ + HF + I^−^, respectively, in good agreement with the experimental values of −48.7 and −37.6 kcal mol^−1^ ^46^. To compare with the experiments, the simulations were performed at a collision energy of 1.9 eV and C_2_H_5_I rotational and vibrational temperatures of 165 and 250 K, respectively. Quasi-classical sampling, which includes ZPE, was used to determine initial conditions for the trajectories, as described previously for the Cl^−^ + CH_3_I simulations^47^. The impact parameter *b* was randomly selected between 0 Ã… and the maximum impact parameter, *b*_max_, which was determined by incrementing *b* until no reaction occurred out of 200 trajectories, which happened at *b*_max_ = 6.0 Å. A total of 1500 trajectories were calculated in the simulations and their atomic-level mechanisms were determined by animating these trajectories. A representative snapshot is given in Fig. 4b.

### Data availability

The experimental data sets generated during the current study are available from the corresponding author upon request.
